# Supplementary material for: Multifunctional Monoclonal Antibody Targeting Pseudomonas aeruginosa Keratitis in Mice
Source: Vaccines (Basel). 2020 Nov 2;8(4):638. doi: 10.3390/vaccines8040638 (PMC7712430; doi:10.3390/vaccines8040638)
Supplement: Supplementary file 1 [file vaccines-08-00638-s001.pdf]

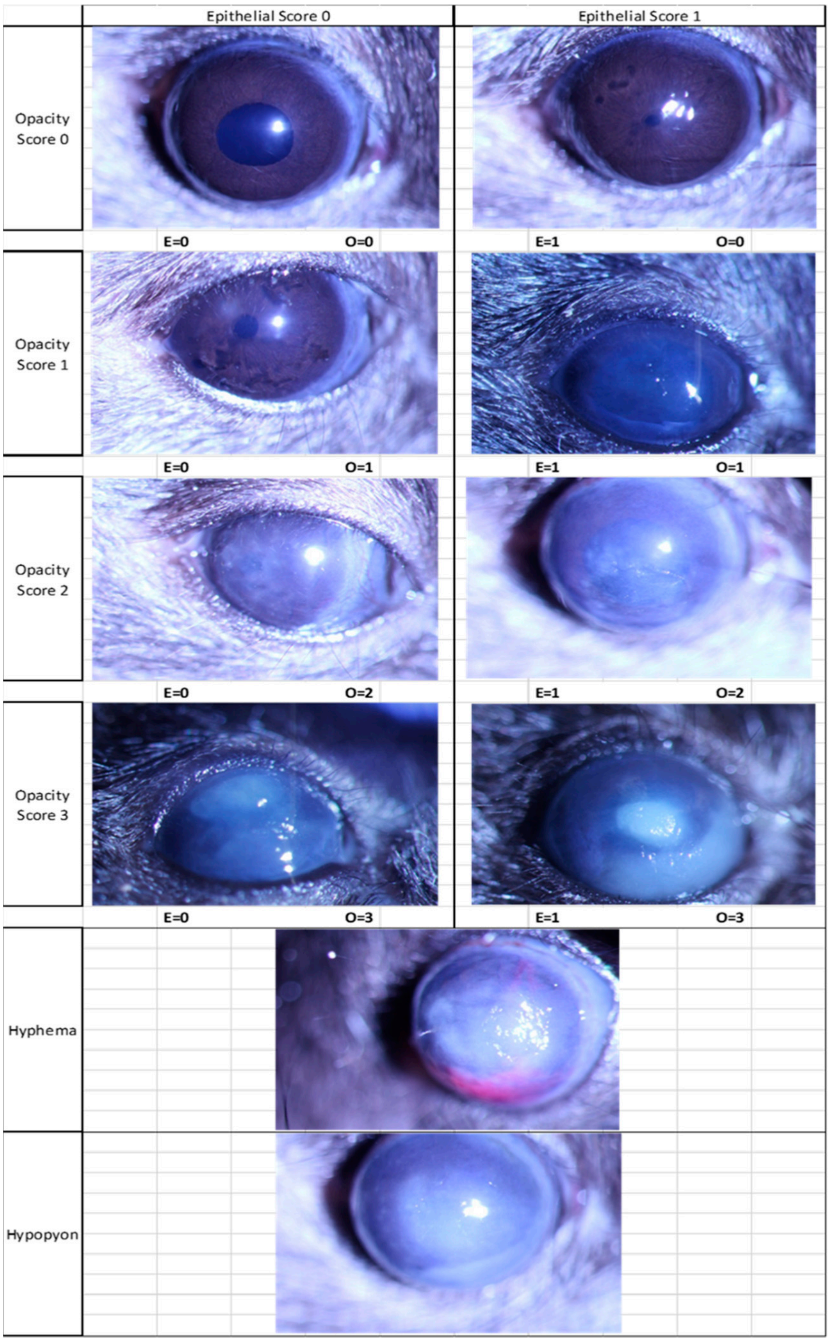

Figure S1. Representative corneal images. Each image provides a sense of the scoring for the Opacity and Epithelial scoring.

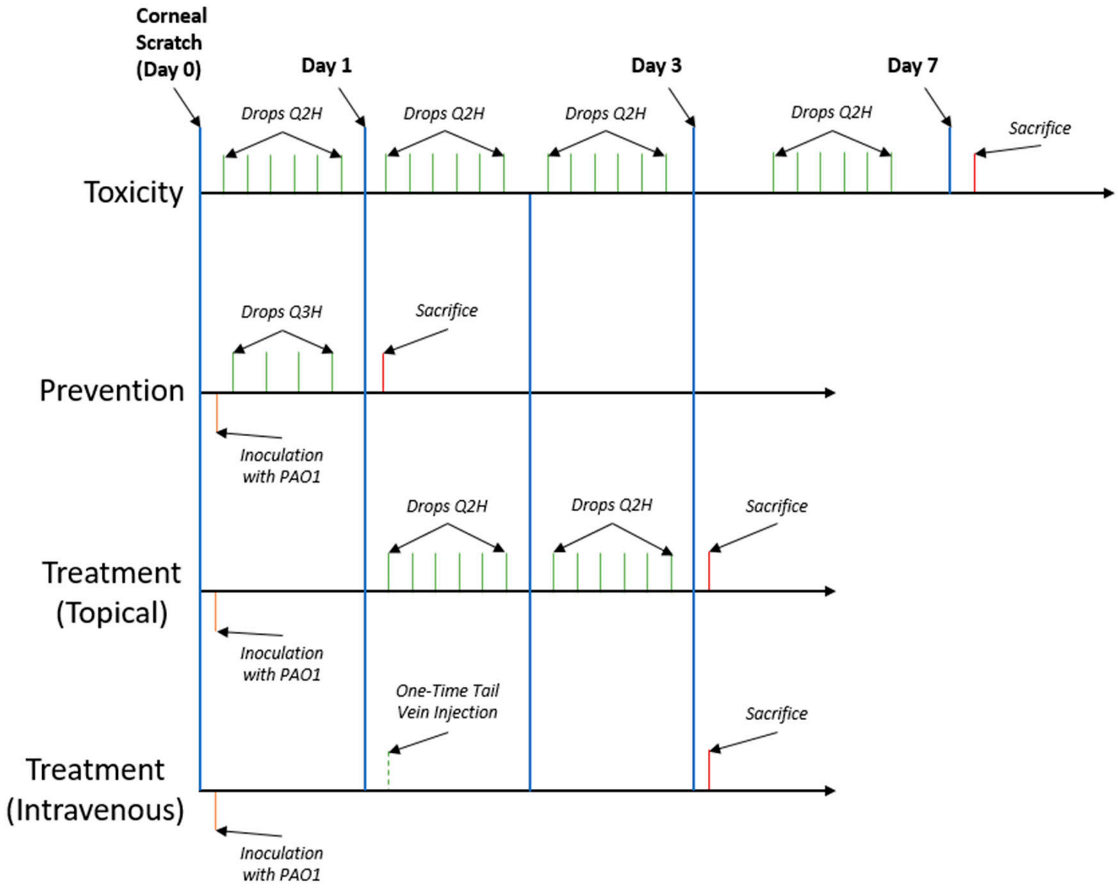

Figure S2. Overall Timescale for Toxicity, Prevention, Topical Treatment, and Intravenous treatment experiments.
